# Supplementary material for: Clonal heterogeneity and antigenic stimulation shape persistence of the latent reservoir of HIV
Source: PLoS Comput Biol. 2025 Sep 15;21(9):e1013433. doi: 10.1371/journal.pcbi.1013433 (PMC12445745; doi:10.1371/journal.pcbi.1013433)
Supplement: S1 Table — The infectivity parameters are listed for an order 11 simulation, but should be increased by one order of magnitude for every one order decrease in the simulation. (PDF) [file pcbi.1013433.s001.pdf]

Parameter	Value	Description	Type	Source
p_{def}	0.002	Probability that an infection event results in a defective HIV-1 provirus.	Fitted	Fitted to recover ratios between defective and intact proviruses during ART of $10-50$ to 1 [28].
p_{mut}	0.33	Probability of a mutation during each infection event.	Literature	Obtained from estimates of the average HIV-1 mutation rate and genome length [52, 53, 54].
p_{L}	0.05	Probability that an infection event will result in a latent rather than active infection.	Fitted	Fitted to recover total levels of HIV-1 DNA per 10^6 PBMC at the beginning of ART measured in patient data [38].
λ_{T}	1.05×10^{10} cells month^{-1}	Replacement rate of uninfected target cells from the thymus.	Literature	3.5×10^8 $CD4^+$ T cells per day at age 20, converted to months [95].
μ_{T}	0.06 month^{-1}	Net decay of susceptible cells. Equivalent to the difference between proliferation and homeostatic death of susceptible cells.	Literature	Value needed to maintain typical size of the $CD4^+$ T cell compartment [94].
β_{EI}	4.0×10^{-13}	Infectivity rate during the first month of infection.	Fitted	Fitted to the rate of viral load increase observed during the first 2 Fiebig stages [92].
β_{AI}	2.76×10^{-13}	Infectivity rate during active infection post immune response.	Fitted	Fitted to recover the viral load observed during Fiebig stages 4 and 5 [92].
β_{ART}	6×10^{-14}	Infectivity rate during ART.	Fitted	Fitted to recover the first stage of viral load decay post ART [38].
μ_A	21 month^{-1}	Death rate of actively infected cells.	Literature	0.7 day^{-1} = 21 month^{-1}, converting previous estimation from per day to per month [49].
γ	1.05×10^5 month^{-1}	Production rate of virions.	Literature	Product of death rate μ_A [49] and the viral burst size $n = 5000$ [50].
c	1.5×10^2 month^{-1}	Clearance rate of free virions.	Literature	Rate converted to per month [51].
λ_f	3 month^{-1}	Inverse of the characteristic lifetime of antigens.	Literature	Converted to per month [46].
γ_f	14.7 month^{-1}	Strength of variability of antigenic environment.	Fitted	Adjusted to recover IUPM fluctuations consistent with observations [59].
ν_L	29.4 month^{-1}	Division rate of latently infected cells.	Literature	Division rate of T cells, converted to per month [46].
μ_L	44.137 month^{-1}	Death rate of latently infected cells.	Fitted	Adjusted to recover average decay in clones with low probabilities of reactivation [59].

Table S1: **Table of parameters used in the model.** The infectivity parameters are listed for an order 11 simulation, but should be increased by one order of magnitude for every one order decrease in the simulation.
