# Supplementary figures and images for: Clonal heterogeneity and antigenic stimulation shape persistence of the latent reservoir of HIV
Source: PLoS Comput Biol. 2025 Sep 15;21(9):e1013433. doi: 10.1371/journal.pcbi.1013433 (PMC12445745; doi:10.1371/journal.pcbi.1013433)

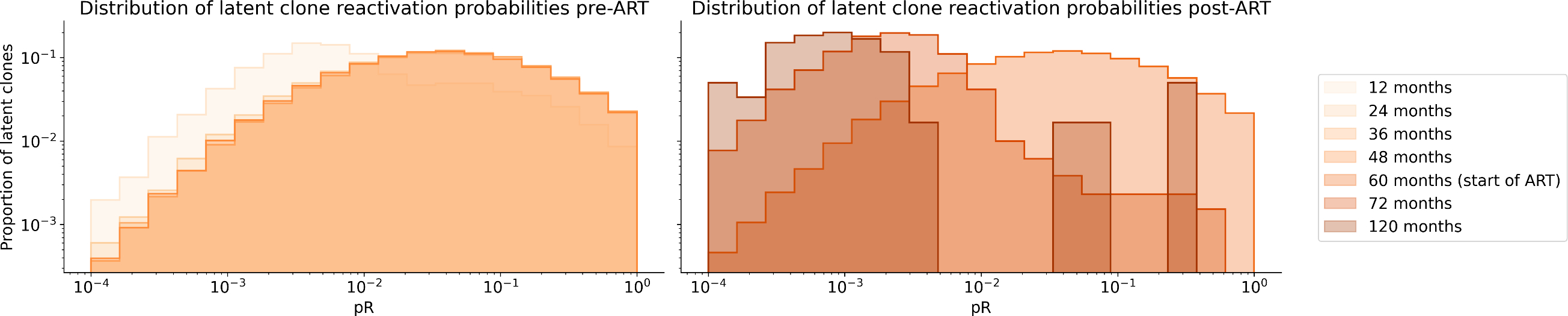

Supplement: S3 Fig — Before ART, the distribution of probabilities of reactivation for clones in the latent reservoir mostly follows the underlying probability distribution used in our simulations (Methods), indicating little selection for or against reactivation during this time. After ART, the distribution progressively shifts towards smaller probabilities of reactivation as more reactive clones are purged from the reservoir. (TIF) [file pcbi.1013433.s004.tif]

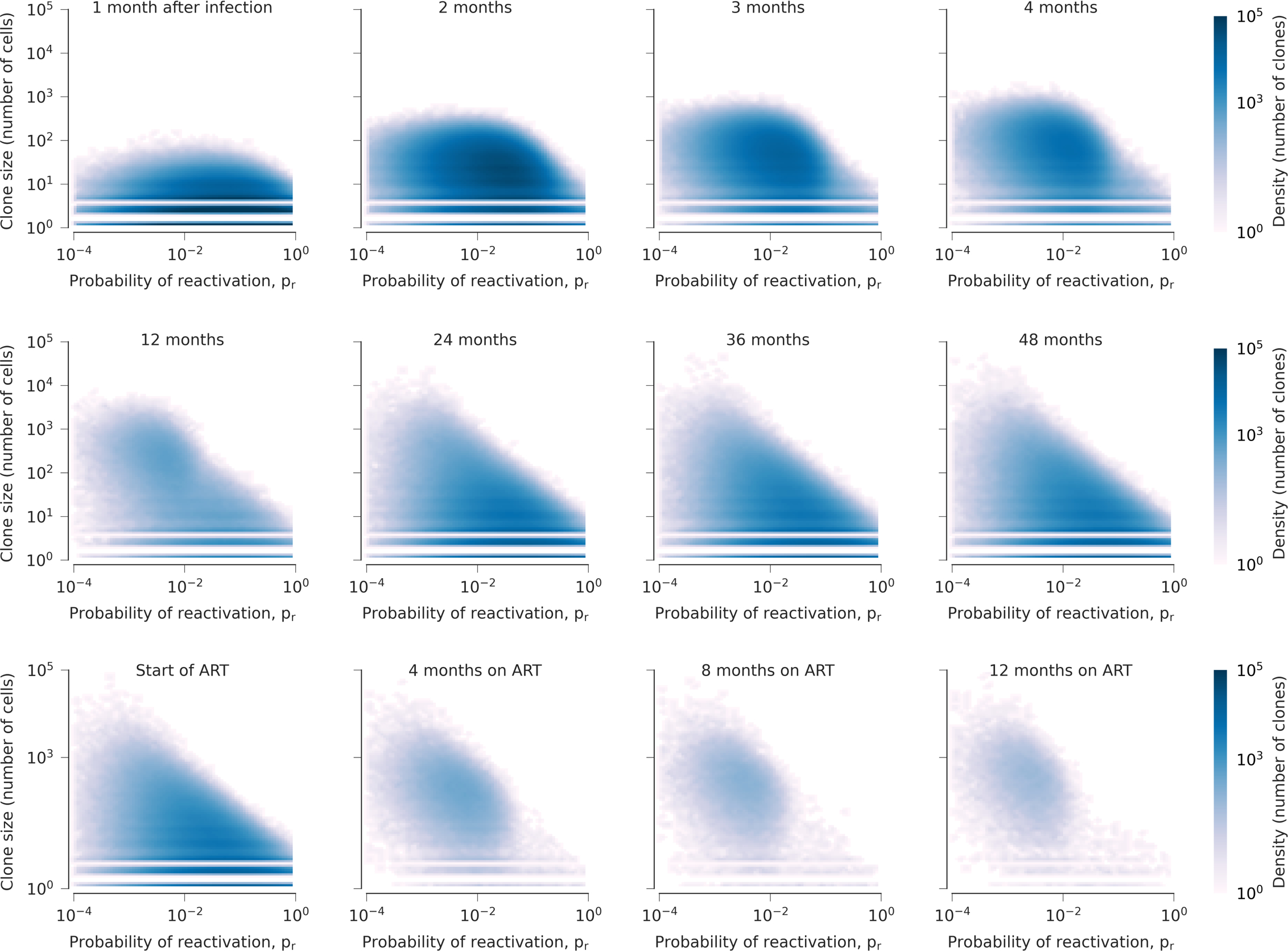

Supplement: S4 Fig — Before ART, clones are broadly distributed in size and reactivation probability. After ART, small clones lost to stochastic fluctuations are no longer completely replenished through new infections. Clones with higher probabilities of reactivation also preferentially eliminated. (TIF) [file pcbi.1013433.s005.tif]

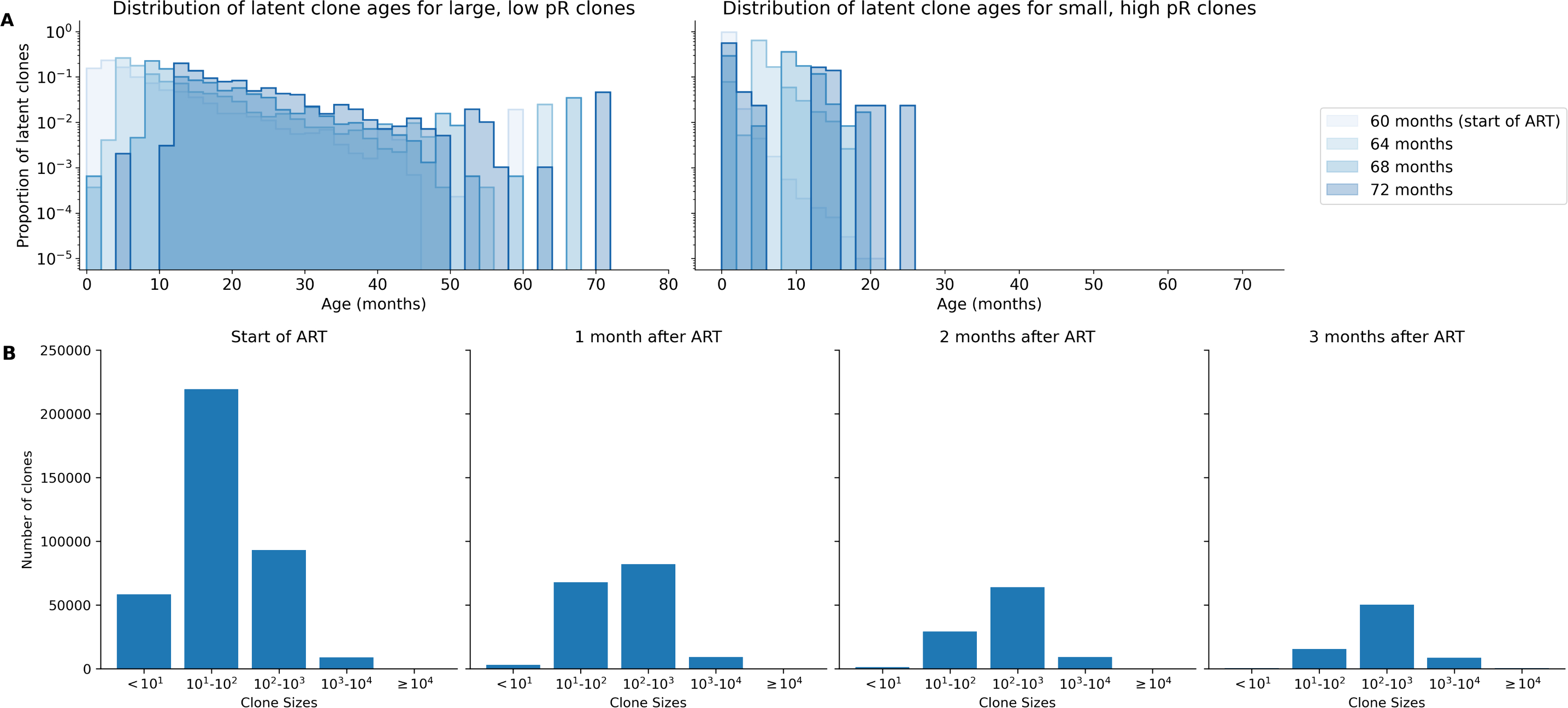

Supplement: S5 Fig — (A) Distribution of ages (in months) for large clones with low probabilities of reactivation (n > 100 cells and pr<0.01) and small clones with high probabilities of reactivation (n < 100 cells and pr>0.01) in one simulation. (B) Distribution of clone sizes from the start of ART until 3 months after ART initiation, showing the rapid elimination of small clones after ART begins. (TIF) [file pcbi.1013433.s006.tif]

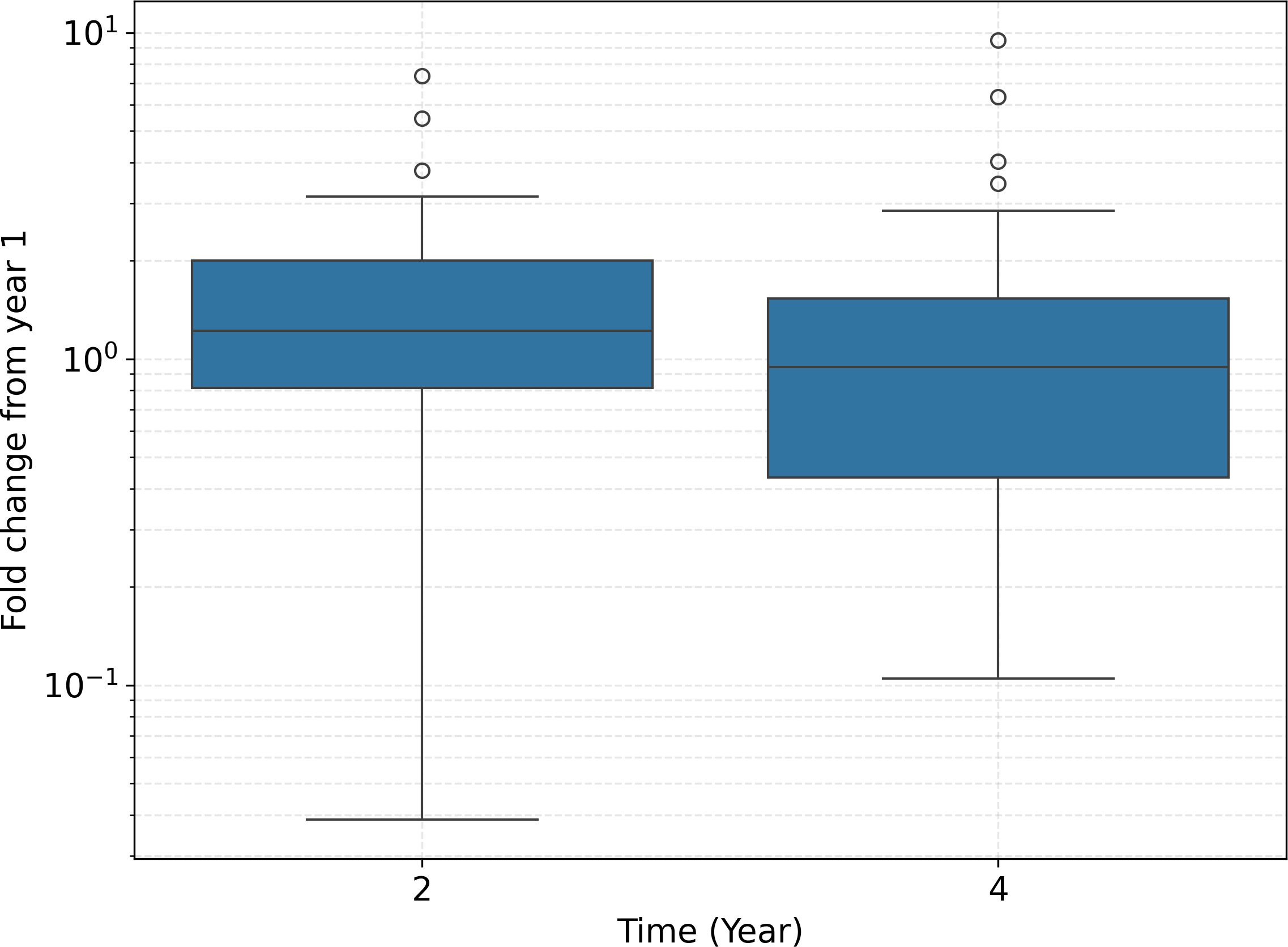

Supplement: S6 Fig — Here we show the distribution of ratios of clone size at two and four years after ART initiation and clone size at one year after ART initiation, specifically for large clones (n > 1000 at one year after ART). On average, clones decrease slowly in size over time. (TIF) [file pcbi.1013433.s007.tif]

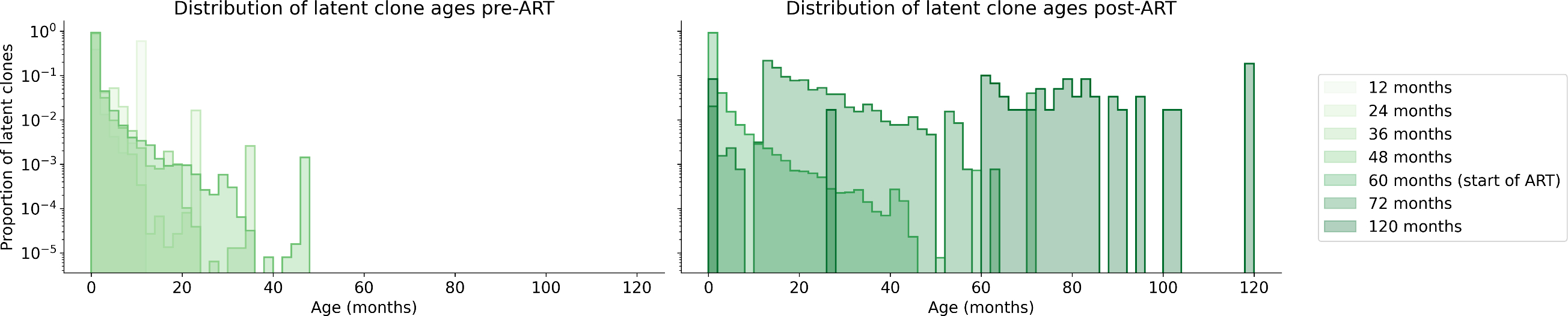

Supplement: S7 Fig — Before ART begins, most clones are young, though there is also a significant spike in the distribution corresponding to clones produced during the early, acute phase of infection. After ART, the clone age distribution shifts toward larger values, with substantial contributions from clones deposited very early in infection. (TIF) [file pcbi.1013433.s008.tif]

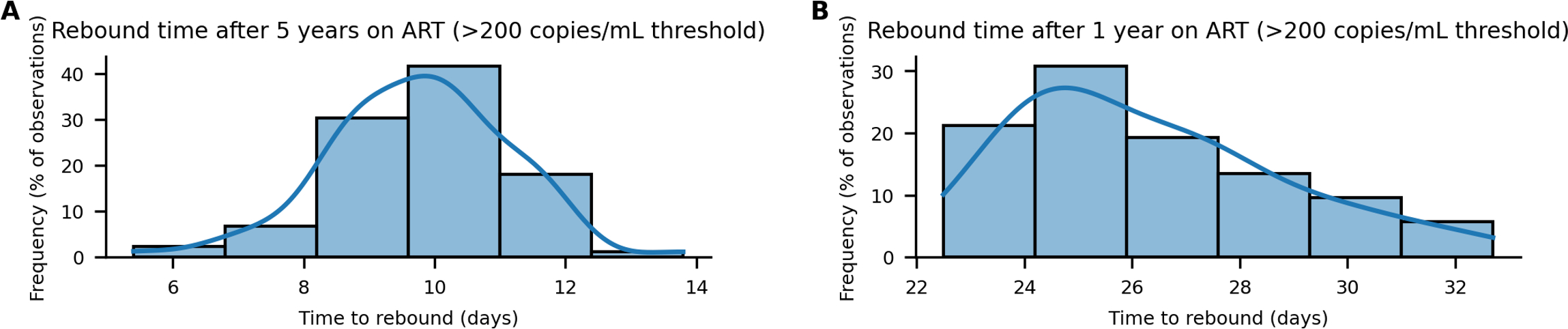

Supplement: S11 Fig — We performed exploratory simulations mimicking analytical treatment interruption (ATI)—a process in which individuals temporarily cease ART in a controlled setting—by restoring the infectivity parameter β to its setpoint value after some time on ART. We defined time to rebound as the first time with viral load >200 copies/mL. (A) In standard simulations, the typical time to rebound after five years on ART was 10 days. (B) For early ART simulations, time to rebound could be much more variable due to the small size of the reservoir. Thus, we simulated ATI at one year post-ART in the early ART case to obtain a tighter distribution in times to rebound. Here, the mean time to rebound for early ART was 26 days, substantially longer than the time to rebound in the standard simulations despite a shorter time on ART. (TIF) [file pcbi.1013433.s012.tif]
