# Supplementary material for: Clonal heterogeneity and antigenic stimulation shape persistence of the latent reservoir of HIV
Source: PLoS Comput Biol. 2025 Sep 15;21(9):e1013433. doi: 10.1371/journal.pcbi.1013433 (PMC12445745; doi:10.1371/journal.pcbi.1013433)
Supplement: Attachment — Submitted filename: 2025-06-13 response.pdf [file pcbi.1013433.s017.pdf]

RESPONSE TO REVIEWERS' COMMENTS
We thank the reviewers for their detailed comments on the manuscript and for their patience as we revised the paper. Due to the graduation of the student who previously led the work and the recruitment of a new graduate student to complete the revisions, it has taken more time than we had originally anticipated to address all comments. Below, we respond to the reviewers' comments and describe our modifications to the manuscript. The reviewers' comments are reproduced in **bold** and our modifications or additions are written in blue.

REVIEWER 1
Noceda and Barton develop a stochastic model of HIV latent reservoir dynamics to investigate potential mechanisms by which clonal heterogeneity develops. Better understanding the development of clonal heterogeneity may provide insight into what types of treatments may be more likely to lead to an HIV cure. The authors show that their simulation fits well to real data and use their model to investigate the dynamics of clones over time during HIV infection pre-and post-ART initiation. While the results appear promising, the authors only show one simulation result, despite the model being stochastic, and do not always clearly show that the model supports the conclusions they make.

Major comments:

1. It appears that throughout the manuscript (Figures 2-5) only one simulation result is shown. However, the model is stochastic, which means that different simulation runs may lead to vastly different outputs. Therefore, the authors should run many different simulations (ideally ≥100 or 1000) and present the results of all the simulations, including a discussion of the variability across simulations, so the reader can better understand how consistent the results are.

We agree that it is helpful to show a range of outcomes from the simulations. With our previous version of the code, running a very large number of replicate simulations would have been extremely computationally intensive. We have since rewritten our simulation code, substantially improving its speed. Note that, during this rewrite, we also modified our simulations slightly to enforce the condition that small clones (<100 cells) have integer numbers of cells only, making our dynamics slightly more realistic. **For this reason, heat maps of clone size distributions (Figs. 2 and 4, Supplementary Fig. 4) will have "bars" at low clone sizes due to the lack of clones with noninteger numbers of cells. We have added a comment in Fig. 2 and in the Methods to clarify this point.** This also allowed us to compute statistics such as the change in the clonal composition of the reservoir and rates of mutation accumulation directly from large-scale simulations. We have therefore updated these figures and removed previous descriptions of simplified simulation procedures in the Methods, since these were no longer needed to obtain our results.

To address this question, we ran 100 repeat simulations at order 9 and plotted the IUPM, RNA copies/mL, and DNA/million PBMC rates of each, shown in an updated **Figure 3**. As expected, due to the stochastic nature of our model, we observe fluctuations between the different

simulation runs over time. The early dynamics after initiating ART are the most tightly constrained and the most reproducible, and the late-time dynamics of latent clones after years on ART are the most variable. This is expected because the dynamics after ART initiation are nearly deterministic, involving very large numbers of active and latently infected cells, etc. However, the dynamics late in infection are driven by the characteristics of a smaller and smaller number of surviving latent clones, which are far more stochastic (**Fig. R1**).

It's also important to emphasize that these simulations give an idea of the range of outcomes that are possible using identical underlying parameters. Variation in viral dynamics between individuals would also be influenced by a host of other factors, including differences in host immune responses (affecting effective rates of replication), duration of infection before ART, therapy adherence and effectiveness, and so forth. Thus, the range of biologically plausible dynamics is certainly broader than what is shown in our simulations. We discuss these factors in a new section in the Results, "Variation in viral dynamics over multiple simulations."

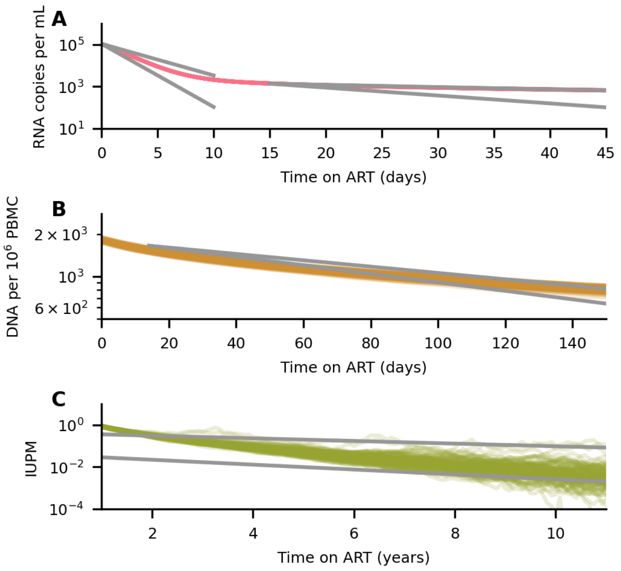

Fig. R1

2. One of the main conclusions, mentioned in the title, abstract, and discussion, is that antigenic stimulation and clonal heterogeneity shape the dynamics of the latent reservoir. In the model, latent cell activation and proliferation are dependent on both the probability of reactivation and the antigen levels. Therefore, it is difficult to differentiate between the effects of antigenic stimulation vs. different reactivation probabilities. To make claims about which of these is influencing LR dynamics, the authors must investigate the impact of each of these components not only together, but also independently. What do the results look like: (1) If simulations are performed where antigenic stimulation is constant across all cells and over time, but cells have different reactivation probabilities? (2) If simulations are performed with a single reactivation probability but fluctuating antigenic stimulation? (3) If both of these are held constant? Do these simulations have a better/worse/similar fit to the data?

Thank you for this suggestion. We ran simulations where the 1) antigenic stimulation was kept constant over time (no fluctuations in antigenic dynamics, and every clone was stimulated by the same concentration of antigen), 2) reactivation probability was the same for all clones, and 3) both antigenic stimulation and the reactivation probability were set to constant values. For all three of these simulations, we see dynamics/statistics that are more difficult to reconcile with the biological data.

In the case of 1), the level of constant antigenic stimulation must be set carefully to avoid either unbounded growth (if the value is too large) or rapid death (if the value is too small) for latent clones. We found that an average concentration of antigen of 14.7 (a.u.) provided the closest fit to post-ART viral dynamics. However, while this choice provided a good fit to the decay of viral RNA and DNA in blood, it led to declines in IUPM that were substantially faster than clinical constraints. This is intuitive because capping the level of antigenic stimulation limits the growth of large clones. Indeed, this was another limitation of the constant antigenic stimulation simulations: the largest clones in constant simulations reached sizes that were an order of magnitude or more smaller than the largest clones in the simulations with variable antigenic stimulation. Furthermore, there were typically a cluster of similarly-sized clones near the maximum in the case of constant antigenic stimulation. In contrast, in the variable case, we tend to observe a small number of very large clones above the “bulk” at intermediate values. The latter case is more consistent with observations in clinical data, where a small number of large clones are often observed to dominate the reservoir (e.g., Lorenzi et al., PNAS 2016). This is also consistent with the findings of (Desponds et al., PNAS 2016), who found that birth-death noise alone was insufficient to produce the broad (power law) range of clone sizes observed in the T cell repertoire (**Fig. R2.1**).

For the case 2), again the choice of the reactivation probability has a strong influence on the results. Very large values result in rapid decay of the LR, while low ones lead to longer-term persistence. As a typical example, we set the reactivation probability to ~1%, which is smaller than the mean reactivation probability in our full simulations but closer to the “bulk” of clones during early ART. Here we observed a modestly faster decline in DNA per million PBMC due and a very rapid drop in IUPM due to the faster depletion of latent cells after ART initiation. Of course, here it is impossible to reproduce the observed relationship between clone size and reactivation probability (Lorenzi et al., PNAS 2016) because this is the same for all clones (**Fig. R2.2**).

For case 3), we see similar outcomes as in case 2), consistent with the more dramatic effects of a constant reactivation probability on the observed dynamics. Here we show results with the same constant antigen concentration as in 1) and the same constant probability of reactivation as in 2). As in case 2), the reservoir decays rapidly and the relationship between clone size and reactivation probability cannot be recovered (**Fig. R2.3**).

Between all three of these simulations, while certain rates (such as the HIV viral load) can fit clinical constraints, others (e.g., IUPM) were more difficult to recover. We have now added a discussion of these alternative models in a new section in the Results, “Alternative models without clonal heterogeneity,” and included figures for cases 1) and 2) above in **Supplementary**

Figs. 9-10. There, we omit discussion of case 3) since this scenario yields results that are qualitatively similar to case 2).

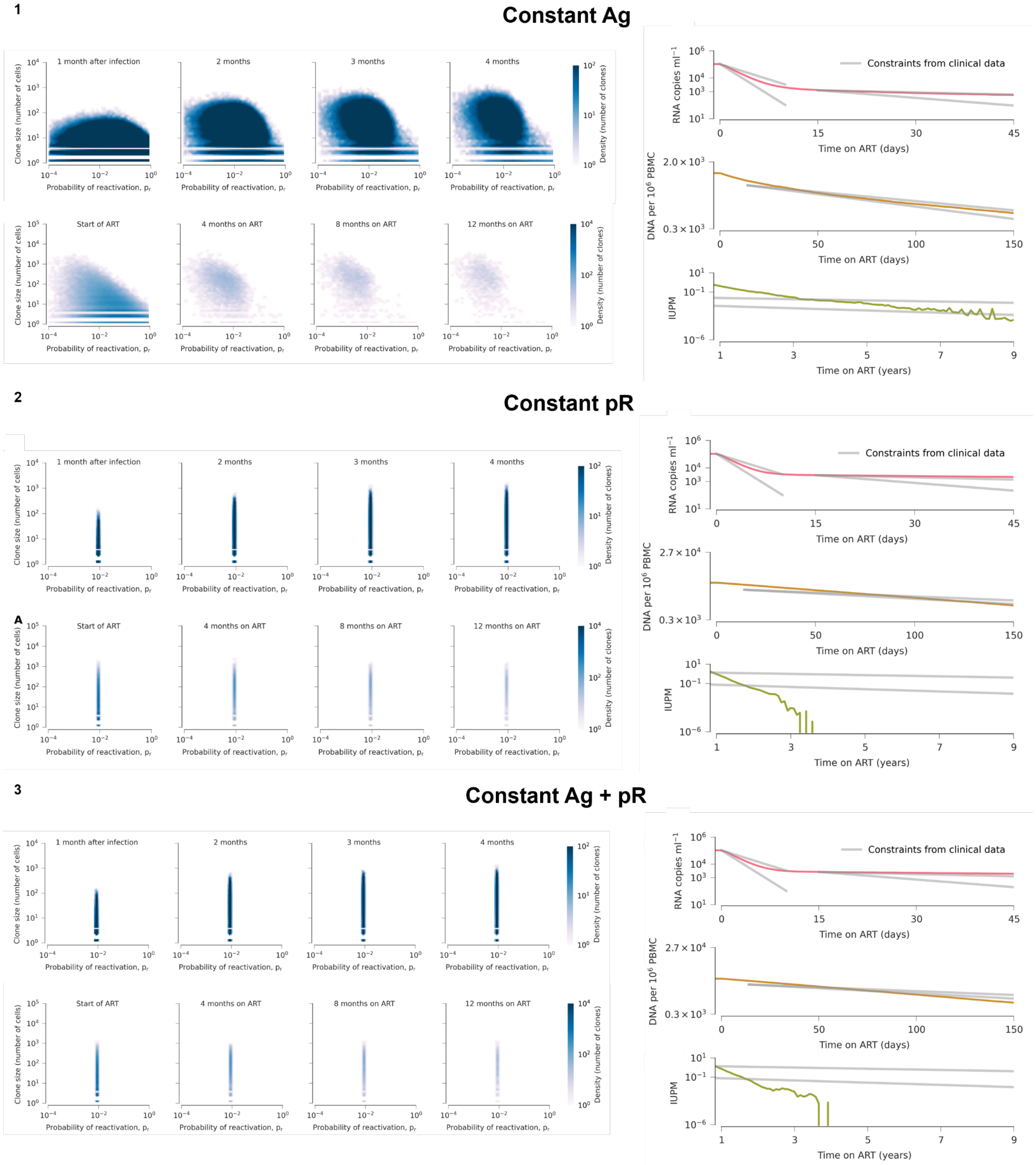

Fig. R2

3. Throughout the results, statements are made that appear reasonable but are not supported with numbers or figures. For example: (1) the viral load drop is primarily driven by the death of actively infected cells, (2) larger clones are slowly eliminated, (3) small clones with high probabilities of reactivation turn over frequently while large

clones slowly fluctuate in frequency, (4) after a year on ART the relative size of large clones changes little over time. It is important to back up these types of statements with numbers/figures from the simulations.

We agree, and we have now produced additional figures to support these points.

To clarify our previous statement, for point (1), virions are only produced by actively infected cells in our model, so the decline in viral load is certainly driven by the death of actively infected cells. We can further break this down by quantifying the two contributions to actively infected cells: new infection events and reactivation of latents. At ART initiation, the number of infection events drops dramatically, as expected, and reactivations begin to supply the majority of actively infected cells, a trend that holds consistently during ART (**Fig. R3.1**).

(2) The gradual elimination of larger clones can already be observed in Fig. 4A. We further analyzed the data to plot the number of clones present for specified clones sizes at 60 (start of ART), 61, 62 and 63 months. Over the course of the first few months after ART initiation, we see that smaller clones (<100 cells) are depleted rapidly in the first month while larger clones (>100 cells) take longer to decay (**Fig. R3.2**).

For (3), we plotted the distribution of clones ages at 60, 64, 68 and 72 months. We define the age of the clone as the difference between the current time (i.e., whichever point in time we are analyzing the data) and the initial infection event that created the new latent clone. The definitions of small/large clones with high/low reactivation rates is qualitative, so to make this precise we defined two subgroups of clones: ones with n<100 cells and reactivation probability > 0.01 (small and high reactivation rate), and ones with n>100 and reactivation probability < 0.01 (large and low reactivation rate). Not all clones fit within these categories, but comparing the ages of clones in these two groups demonstrates the point that we intended to make. For the large clones, we see a wider distribution of clone ages across all four months, with this distribution gradually shifting towards older clones. The small clones have a tighter distribution that is strongly concentrated at younger ages. This is also consistent with recently published findings from Sambaturu and collaborators (Cell Rep. 2025), who observed that longer-lived cells harbored viruses deposited earlier in infection while shorter-lived ones hosted more recent viruses (**Fig. R3.3**).

(4) To show the relative change in large clone frequencies after extended ART, we plotted the fold change in size for large clones comprised of over 1000 cells. The size of large clones at years 4 and 2 were divided by their size at year 1 post-ART and this change was visualized as a box plot. There is significant overlap in the clone size differences between year 4 and 2, and the distribution is centered around a fold-change of slightly less than one, indicating that the clone sizes decreased modestly but typically without large fluctuations after a year or more on ART (**Fig. R3.4**).

We have added these as the following Supplementary Figures: **Sup. Fig. 2** (Point 1), **Sup. Fig. 5** (Points 2 and 3), and **Sup. Fig 6** (Point 4). We have also discussed this supporting evidence in the following two sections of the paper: “Kinetics of plasma viral load, HIV-1 DNA, and the

inducible viral reservoir after ART initiation” (**Sup. Fig. 2**) and “Long-term clonal dynamics in the latent reservoir” (**Sup. Figs. 5-6**).

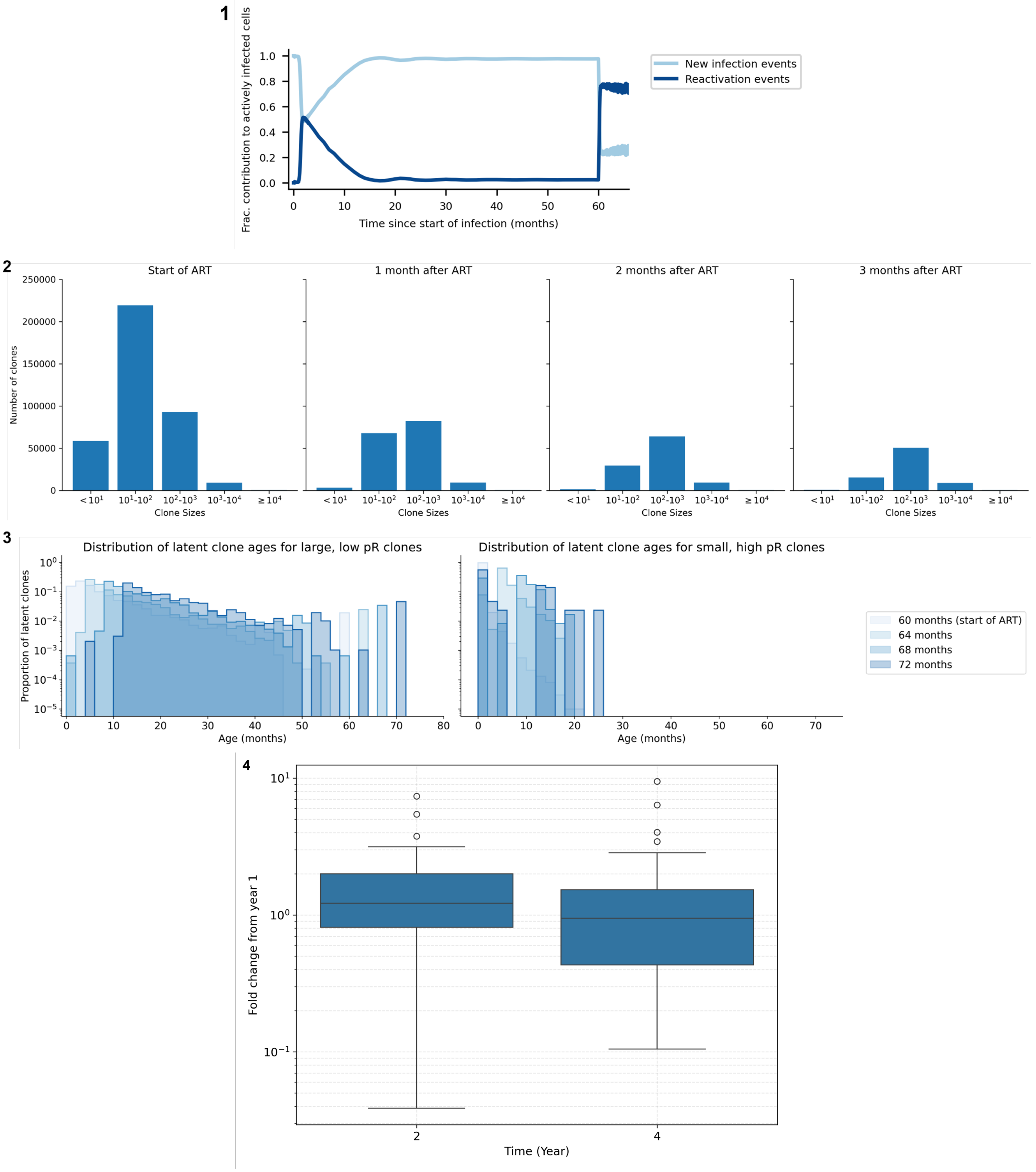

Fig. R3

4. You mention that "even though we find that the largest clones (with small probabilities of reactivation) are chiefly responsible for LR persistence, they may not be the typical first source of outgrowing viruses during rebound." Can you simulate this to see if your hypothesis is true?

Thank you for this suggestion. While our model has not been specifically fitted to reproduce viral rebound statistics, we can simulate a simple ATI scenario in our model. We cover the results of those simulations in response to Reviewer 3 below.

This particular question can also be answered more comprehensively in another way. First, note that rebound will be driven by actively infected cells produced around the time of treatment interruption. Thus, we can analyze which clones contribute the most to reactivation during ART, as those will be the ones that drive viral rebound. We divided the clones into bins based on size (<10 cells, 10-10^2 cells, 10^2-10^3 cells, 10^3-10^4 cells, and >10^4 cells) and counted their contributions to reactivation across simulations. For our full order 9 simulation, we see that the smaller clones (bins 1 and 2) are responsible for a little over 90% of the total reactivation events during active infection (i.e., before ART). However, after ART initiation, the intermediate-sized clones in bins 2 and 3 make the dominant contribution towards reactivation. The largest clones (>10^3 cells) contribute far less to reactivation, and thus they are relatively unlikely to be the source of rebound viruses (**Fig. R4.1**). A similar phenomenon can also be observed for the early ART case, though here all clone sizes are shifted toward smaller values due to the smaller size of the LR (**Fig. R4.2**). We have included just the plot of clone size counts contributing to reactivation during ART for the full simulation as **Supplementary Fig. 12** and discuss this result in the "Factors underlying long-term LR persistence" section of our results. We chose to omit the other three plots shown here as we focus on discussing the full simulation in this section, and highlight the importance of clones produced during ART as the major contributors to rebound. Additionally, we have added **Supplementary Fig. 11**, which is discussed in our new methods section titled "Simulation of analytical treatment interruption" which describes the ATI simulations we ran to generate these figures.

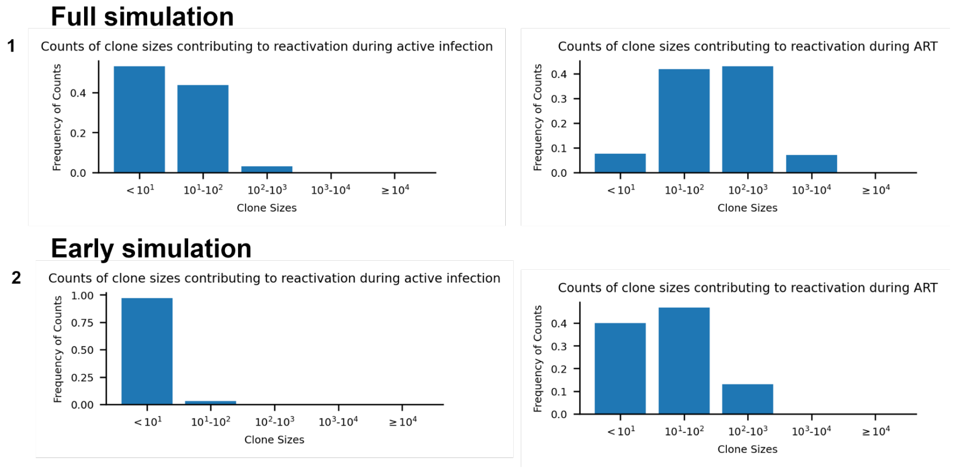

Fig. R4

5. Related to clone size, reactivation rate, and age over time. It would be interesting to see the plots in Fig 2 and Fig 4 for times between 4 months and 60 months, and after 12 months on ART. It would also be helpful if all these plots were in one figure so that it is easier to see the progression over time. You also say that 1 year post-infection, the clones on average are young and likely to reactivate – how does this change throughout infection? It would be nice to include as a figure the distribution of clone ages at different times of infection.

Thank you for this suggestion. We have now generated plots to show these distributions (**Fig. R5**). **Supplementary Fig. 4** now shows this progression in the clone size distribution over time. **Supplementary Figs. 3** and **7** show the distribution of clone ages and probabilities of reactivation over time. We discuss these results across several sections, some of which are new. For **Supplementary Fig. 3**: “Kinetics of plasma viral load, HIV-1 DNA, and the inducible viral reservoir after ART initiation” and “Processes driving latent cell proliferation and death”, **Supplementary Fig. 7**: “Dynamics of clone age pre- and post-ART”, and **Supplementary Figure 4**: “Long-term clonal dynamics in the latent reservoir”. One can see that young clones dominate pre-ART, but that these shift toward older values during long-term ART (**Fig. R6.1**). Similarly, the distribution of probabilities of reactivation shifts from a peak just under 10% early in infection (due to our choice for the underlying distribution) toward much smaller values post-ART as clones with high probabilities of reactivation are eliminated (**Fig. R6.2**). This is also consistent with some of the points raised in comment 3 above.

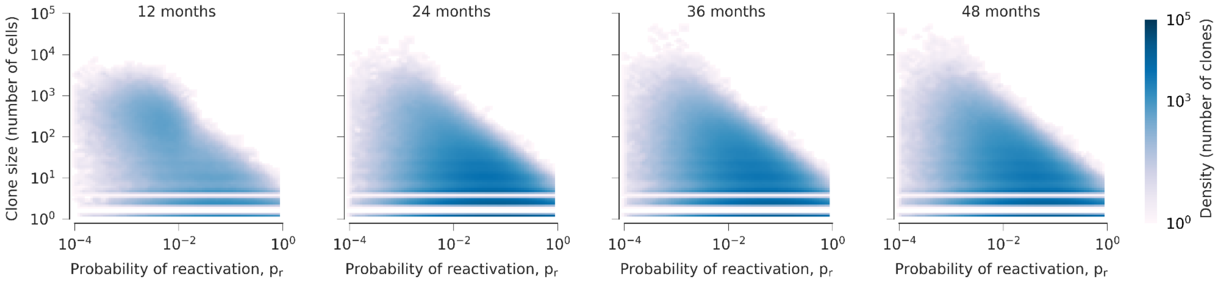

Fig. R5

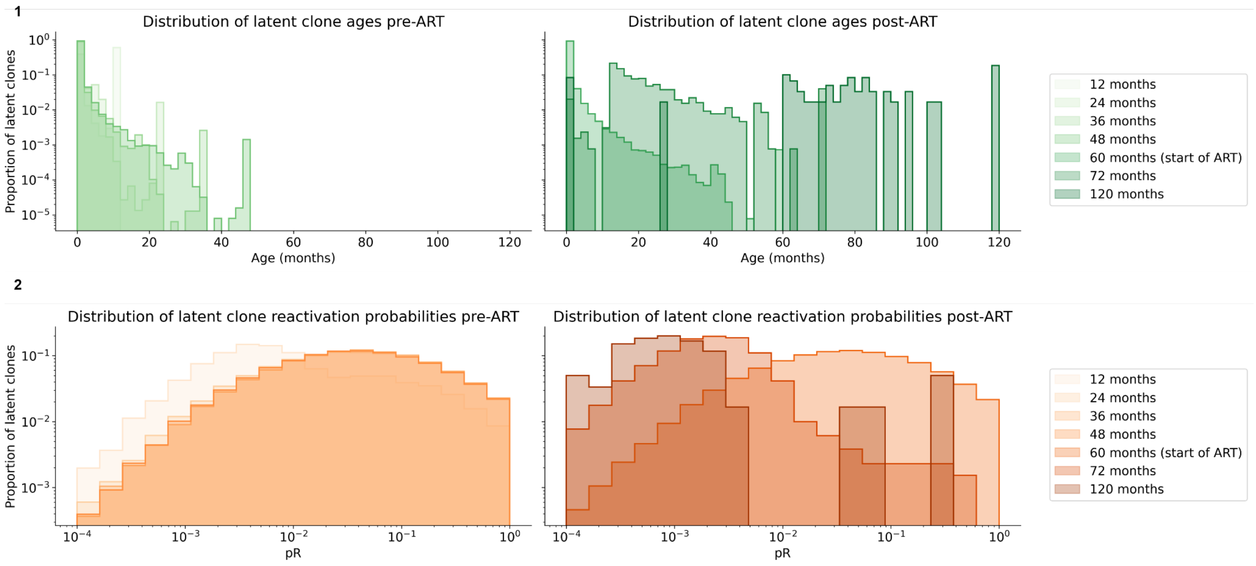

Fig. R6

6. In Fig 3, can you include the kinetics of plasma viral load, HIV-1 DNA, and the inducible viral reservoir pre-ART as well as post-ART?

We've now added a new **Supplementary Fig. 1** (**Fig. R7** below) that shows the dynamics of actively infected cells, virions, and latently infected cells from the start of the simulation up to 5 years after ART initiation (Fig. R7). The primary focus of our work is on the dynamics of the latent reservoir, and thus we have not attempted to fit detailed viral kinetics prior to ART beyond those already described in Methods. Some features of the simulations (e.g., oscillations in actives and virions pre-ART) are not fully realistic but could be addressed in future work with an expanded model that incorporates more realistic dynamics for uninfected T cells and the immune response. This figure is discussed in the section titled "Kinetics of plasma viral load, HIV-1 DNA, and the inducible viral reservoir after ART initiation".

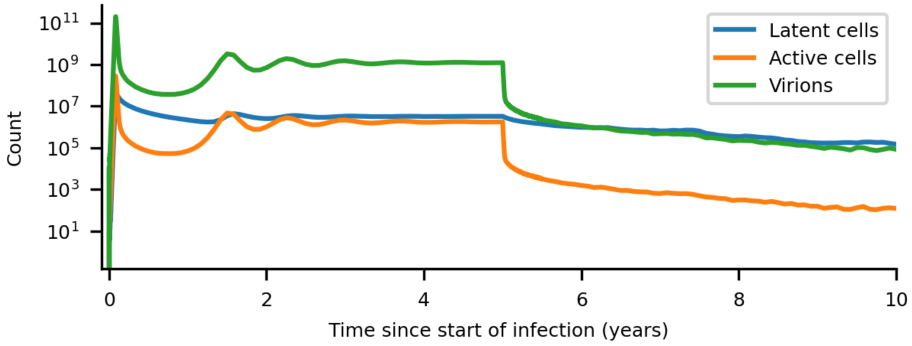

Fig. R7

7. The discussion of how the model output changes with respect to the lognormal distribution parameters should go in the main text rather than the methods. A supplemental figure supporting the statement would also be helpful.

We have now moved this content into a new section in the main text, “Choice of reactivation probability distribution and the role of heterogeneous reactivation rates.” In this section, we also discuss the dynamics when probabilities of reactivation are equal across all clones. This provides an opportunity to discuss **Supplementary Fig. 9** and related results, such as the accelerated decay observed in clones with high probabilities of reactivation and how this affects viral dynamics.

Minor comments:

1. Why does Figure 3C start at 1 year post-ART when the others start at 0 years post-ART? I think it would make more sense to start all of them at 0. (Or, as mentioned above, also look at pre-ART levels.)

This is to compare with studies of the long-term decay of the inducible reservoir, which, to our knowledge, have typically (though not always) examined IUPM changes after >1 year on ART (e.g., Finzi et al., Nature Medicine 1999). In our model, we observe a drop in IUPM early after ART initiation that is more rapid than the long-term decay rate. After 1-2 years on ART, the decay of IUPM in our simulations aligns with the rates in clinical literature.

2. If you want to describe why you’re seeing the trends in Fig 3, then Fig 4A should be referenced there as well.

This is an excellent point. We have now referenced **Fig. 4A** as appropriate when discussing the trends that we observe in **Fig. 3**.

3. Could you put the bars for Fig 4B side-by-side so it is easier to see the difference in the accumulated number of mutation for 1 vs. 5 years? (e.g. make the years two different colors)

We have now placed the bars for the accumulated mutations as requested so that the distributions can be more readily compared. Note that these mutation results were now obtained directly from full-scale simulations rather than averaging over simplified simulations to estimate mutation accumulation, and thus the distribution is not identical to the previous plot.

4. In the equations, what is W?

W represents the Wiener process or Brownian motion, which is a stochastic contribution to the equations in the Methods. These fluctuations are due to either finite numbers of components (virions, latent cells, etc.) or, in the specific case of the antigenic noise, randomness in the environment. dW is equivalent to the Gaussian white noise eta in Eq. 2.

REVIEWER 2

The manuscript builds a stochastic differential equation model of HIV infection, involving HIV-1 virions, uninfected CD4+ T cells, productively infected activated CD4+ T cells, and latently infected resting CD4+ T cells. Clones of CD4+ T cells are defined as latently infected cells with identical T cell receptors, integrated proviruses and integration sites. When exposed to their corresponding antigen, a clone may reactivate with a certain probability. If reactivation does not occur, exposure to the antigen results in clonal expansion. This dynamic between antigen stimulation and clonal expansion leads to several interesting predictions regarding the dynamics of the latent reservoir, including a progressive increase in the number of clones with low reactivation probabilities and large clone sizes over time.

The authors have carried out the simulations with a realistic number of CD4+ T cells where possible, and made clever simplifications where not possible. The simulations agree quantitatively with experimentally observed dynamics of HIV-1 days after the initiation of ART, and IUPM years after the initiation of ART. The model also dives into the possibility of HIV replication and/or evolution in the LR, and suggests that sporadic replication during ART is consistent with experimental data, but persistent replication is not.

Overall, the paper is well written, and presents a compelling case for the relationship between clonal heterogeneity and antigen stimulation, and the dynamics of the latent reservoir. However, a few questions remain, that need to be addressed.

Questions

The authors state that the model of clonal proliferation in response to antigen stimulation implemented here differs from previous studies. However, the biological rationale or justification for the choices made here seems to be missing. It would be great if the biological rationale can be provided explicitly, along with relevant references.

Thank you for this question. In our section titled "Stochastic model incorporating LR heterogeneity", we now cite experimental work from Bosque et al. (PLoS Pathogens 2011) and Moskovljevic and collaborators (Immunity, 2024) that shows that antigenic stimulation can drive reactivation. Furthermore, Moskovljevic et al. show that this process is stochastic, with high levels of HIV-1 transcription only observed in rare cases, and Bosque et al. showed that reactivation during homeostatic proliferation is rare. These observations are consistent with our modeling assumptions, and we have now cited this work in the description of our model.

The model description in the methods seems to skip several steps between the explanation and the equations. In fact, some of the variables don't seem to have explanations at all, such as W_(L_i) in equation (1), D in equation (3). It would be great if all the steps could be worked out, at least in the supplement.

Thank you for this comment; indeed, the previous Methods were sparsely described. We have now added more intuitive descriptions for the equations presented there. We have also slightly

expanded the mathematical description and added a key background reference for general models of this type.

Paragraph 3 of the introduction contains several references (refs 15 to 22) in which the findings are relevant to Figures 2, 4, and 5. It would be good to compare the model predictions with the data in these papers, either directly as has been done in Figure 3, or at least in the discussion.

We have endeavored to discuss the parallels between our model and these (and other) references more fully throughout the text and in the Discussion. In general, there is good agreement between prior work and outcomes in our simulations. To describe why these references were included in more detail, we give the subsequent reasoning for each figure. **Figure 4** is supported by reference 15, stating that background antigenic stimulation can drive the proliferation of larger, less reactive clones in the latent reservoir. References 17-18 were additionally used to support this figure as they suggest that clones with lower reactivation probabilities may have provirus integrated at more transcriptionally silent sites, a potential connection that was also highlighted by Reviewer 3. Refs. 15, 16, and 19-21 further highlight the role of antigenic stimulation and homeostatic proliferation in reservoir growth and persistence, now quantified in **Supplementary Fig. 8**. For **Figure 5**, we used reference 22 to support our finding that the size of the latent reservoir was much smaller after early ART compared to the standard simulation.

Refs. 18 (Einkauf et al., Cell 2022) and 21 (Simonetti et al., PNAS 2016) are the most in tension with our model, since they find some large clones that are transcriptionally active or contribute to active viral replication. In our model, the largest clones are typically more quiescent. We discuss this point in the Discussion, which may highlight ways to further improve the realism of our model.

Cho et al. 2022 [1] found that the clonality of the intact latent reservoir increases and its diversity decreases over time. It would be good to cite this paper and compare and/or comment.

Thank you for pointing this out. While a full quantitative comparison is beyond the scope of the present work, the findings of Cho et al. are certainly broadly consistent with our model. We have now cited this paper in the Introduction and our section titled “Seeding of the reservoir and clonal proliferation pre-ART.”

In the results presented in the main manuscript, the time of ART initiation has not been mentioned, except in the results relating to early intervention or elite control. The Methods section states that ART initiation occurred in the simulations at 60 months post-infection. It would be good to state the time of ART initiation in the simulations explicitly in the results section. A rationale needs to be provided for picking 60 months post-infection as the time for ART initiation.

We have now included this number in the "Kinetics of plasma viral load, HIV-1 DNA, and the inducible viral reservoir after ART initiation" section of the text as requested. The precise timing of ART initiation is not an essential factor in our model (as long as this is not extremely early, as in the early ART scenario that we examine in the paper), but this number is roughly consistent with published studies of the typical delay between HIV-1 infection and diagnosis (median ~3 years). We have now included references for this time between infection and diagnosis in the paper. As an example, in **Fig. R8** below we also show the outcome of a typical simulation with ART initiation at 3 years after infection, with qualitatively similar dynamics to those obtained when ART is started after 5 years.

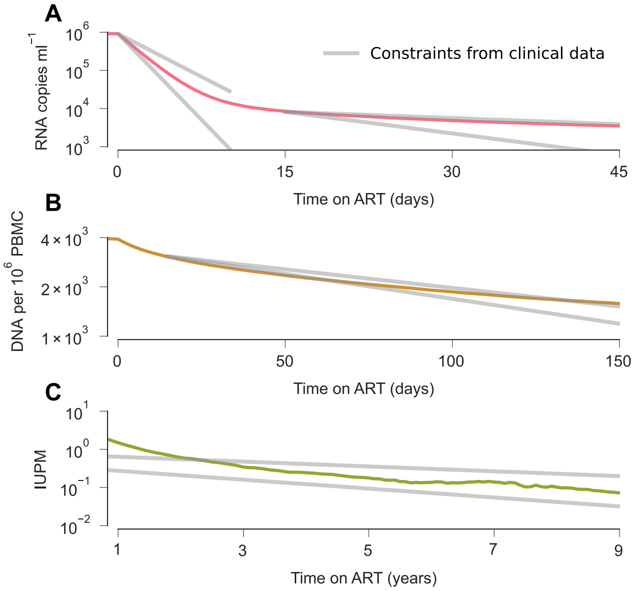

Fig. R8

Figure 3 includes constraints from clinical data, and the simulation results are generated for the scenario when ART was initiated 60 months post-infection. This needs to be compared with the time when ART was initiated in the clinical data. It would be good to comment on how the time of ART initiation may affect the phenomena being modeled, and the dynamics of the latent reservoir.

We agree that it would be ideal to compare the dynamics that we observe in Fig. 3 (and in other parts of the paper) with precisely matched clinical conditions. At present, we are not aware of data that reports clinical viral dynamics with this level of specificity. We have now added a paragraph in the Discussion to highlight this limitation in our analysis and to comment on how it may affect the interpretation of our results. Specifically, since small clones equilibrate rapidly after acute infection while very large clones require some time to grow, we expect that viral dynamics that depend on 1) the existence of very large clones or 2) a very broad distribution of clone sizes would be most sensitive to misspecification of the timing of ART.

Minor questions

In the first paragraph in the Mathematical model (Methods), the text says “HIV-1 virions can infect target CD4+ T cells, and upon infection, a small fraction will result in defective cells due to …”. I believe the authors meant defective proviruses, right?

Yes, you are correct. Thank you for pointing out this error. We have corrected the text to read “cells with defective proviruses” instead.

The section “Modeling seeding of the reservoir and ART initiation” mentions Fiebig stages 1, 2, 4 and 5. What about stage 3?

Thank you for pointing out this omission. Fiebig stage III is near peak viral load, and thus we had omitted this from our description of the rise/fall in viral load. However, this question highlights that the most important inputs into the model are not the specific Fiebig stages (defined by certain outcomes in clinical assays) but rather the general arc of increase and decrease in viral load. We have therefore edited the text to refer more qualitatively to “early” and “set point” viral load as guides for our model.

References
[1] Cho, Alice, et al. "Longitudinal clonal dynamics of HIV-1 latent reservoirs measured by combination quadruplex polymerase chain reaction and sequencing." Proceedings of the National Academy of Sciences 119.4 (2022): e2117630119.

REVIEWER 3
Noceda and Barton have put together a beautiful modeling study that is the first to explore a fundamental component of HIV latent reservoir biology, that there is a continuum of proliferation and reactivation rates/probabilities across various sequence clonotypes. This is an excellent contribution and helps inform how the reservoir persists as well as why we see the type of data we see in experiments.

Sincerely,
Daniel Reeves, PhD

I had a few larger questions/comments:

1) The manuscript covers a lot of ground in a relatively short work, I do think it could be advantageous to focus the paper slightly and come to more concrete conclusions in several places. Just as a couple examples, it isn't clear exactly what features of the experimental data are missed in a model without clonal heterogeneity (maybe something like Fig S1?), and it isn't clear what fraction of latent cells you expect are generated via replication vs Ag stimulation vs homeostatic proliferation and what fraction are cleared via homeostatic death vs reactivation. I think writing a bit more like this would make the paper more enticing to experimentalists in this field.

Thank you for this question. Simulations in response to some other comments have also allowed us to explore the importance of clonal heterogeneity in different ways (see in particular comment 2 from Reviewer 1). Generally, we find that differences in reactivation probabilities are very important in our modeling framework: models with constant probabilities of reactivation are very difficult to fit to the long-term decay of IUPM observed in clinical data. Without heterogeneous antigenic stimulation, we observe fewer fluctuations in the latent clones, including a lack of very large clones that stand well above “bulk” values. We also found it more challenging to reproduce observed IUPM decay in this scenario.

We have also now quantified the different ways in which latent cells are produced and how they die over time in our model. Interestingly, we find that the production of new latent cells through new infection events only dominates during the very earliest stages of infection; after that time, homeostatic proliferation and antigenic stimulation contribute more to the growth of the latent compartment than infection, even before ART begins (**Fig. R9**, added as a new **Supplementary Fig. 7**). While initially surprising, this result is actually consistent with the long-term persistence of the LR even in individuals who began ART shortly after infection. In our model, reactivation is never the primary driver for latent cell death, even during acute infection. Nonetheless, reactivation plays an essential role in shifting the distribution of latent clones over time from ones that are more prone to reactivation to ones that are more quiescent. We have added **Supplementary Figure 7** and discussed these results in the new section titled “Processes driving latent cell proliferation and death.”

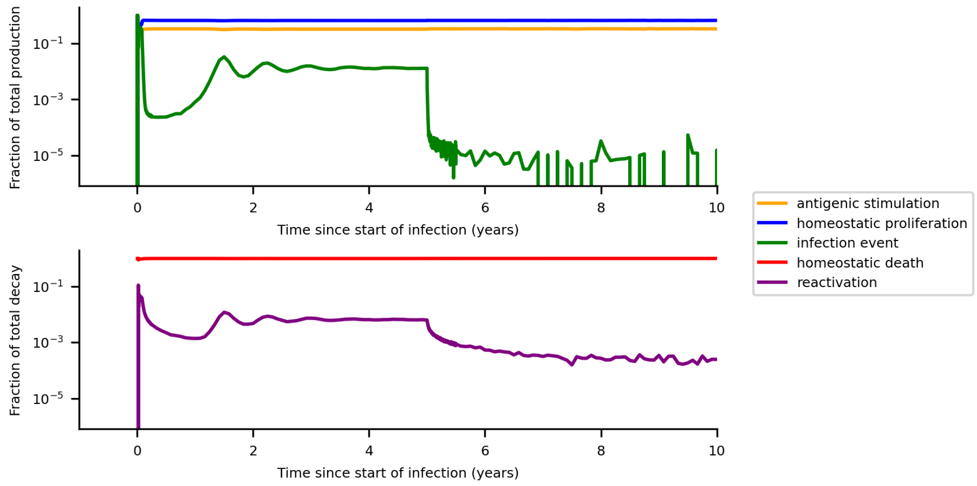

Fig. R9

2) It seems biologically plausible that proliferation and reactivation are linked together, which if I understand correctly is not considered in this version of the model? It could be important to show whether or not this interaction changes any conclusions (I guess somehow correlating the draws of p_r_i and f_i?)

This is a very interesting suggestion. In our model, we would tend to view the f_i as set by the antigenic environment, and thus independent from reactivation. We have assumed that homeostatic proliferation is also unlikely to result in reactivation, consistent with past

experimental work (Bosque et al., PLoS Pathogens 2011). While we believe that an extended analysis is beyond the scope of the current work, this idea could be incorporated by allowing the homeostatic proliferation and/or death rates to vary between clones. It would also be very interesting to explicitly model proviral integration into HIV-specific T cell clones, where the connection between viral replication and antigenic stimulation is explicit.

3) It's been very hard to find the clones that eventually actually rebound in ATI studies, could you possibly do a rough calculation here for how many cells one would need to sample to start finding those rarer/reactivation competent proviruses in a person on ART for years (started early vs late)?

Thank you for this point, which touches on a similar question as in the comments from Reviewer 1. The figures we included in response to their point help support our explanation here. We tracked the contributions that clones of different sizes make to reactivation during long-term ART. Since viral rebound is likely to be driven by reactivation events around the time of treatment interruption, clones that contribute the most to reactivation will be those that are most likely to drive rebound. We found that intermediate-sized clones (10-1000 cells) contribute to the great majority of reactivation during ART (see new **Supplementary Fig. 12**). Based on the average size of the latent reservoir following ART, this suggests that we would need to sample around 8,500 intact proviruses before sequencing the specific clone that initiated rebound in a typical case. For early ART simulations, both the size of the reservoir and the size of the clones that contribute most to reactivation are smaller. This results in an estimate of around 400 intact proviruses that would need to be sampled before identifying the source of viral rebound. We have now added these rough estimates to the text in the section “Factors underlying long-term LR persistence”, along with other Supplementary Figures and text describing our exploratory ATI analyses.

Minor comments:

-when you say genetic material incorporated... you could be a little more precise and say something like viral RNA is reverse transcribed and resulting DNA integrated into the cell's genome, also would give a chance to define provirus here

Thank you for this suggestion, we have now edited the text to be more precise.

-would maybe restate "many remain capable" because the majority are defective, and later you mention the extra rarity of reactivation competent cells

Yes, this is a good point. We have now edited this to specify that “While many latent proviruses are defective, some remain capable of reactivation, …”

-double check whether those refs 9-11 say 1 in 1e4 T cells vs CD4+ T cells?

Thank you, we have clarified that this refers specifically to $CD4^+$ T cells.

-the para on biological mechanisms might be expanded just a little to make sure a reader gets that there are a bunch of possible mechanisms operating, from homeostasis (cite Chomont), Ag proliferation (TCR linked, cite Lillie Cohn and Simonetti papers), longevity (cite Lisa Frenkel) and integration site transcriptional effects (probably should cite wagner/maldarelli Science 2014 as well as newer work on silencing from Matthias Lichterfeld/Xu Yu among others here)

Thank you for these suggestions. We have now added more references to this discussion and mentioned differences in HIV-1 transcription, which we had omitted in the prior version of the paper.

-similarly, it would be good to write a little more to introduce the system you are trying to model: a multi-clonal population where certain HIV seq clones are integrated into certain TCR seq clones

Thank you for this suggestion; we have now expanded our description of the model to make this aspect of the work more clear.

-fig1, love this figure, but one part that isn't clear visually is whether the susceptible cells are clonal (I think they are? and power law unevenly at that)

This is a very good point — we were not entirely clear in our previous description of the model, and indeed it could be interpreted as modeling the susceptible T cells as all having unique TCRs and responses to antigen. However, our approach here is simpler: we treat all the susceptible T cells the same, reserving antigen-driven dynamics only for latently infected cells. Each new latent clone has an independent antigenic noise term, and thus effectively a different TCR. We have now clarified this assumption both in the text and under **Fig. 1**. We believe that in future work it would be very interesting to model susceptible T cells in more detail as well, though this is also highly computationally intensive.

-minor bookkeeping but the early Perelson HIV models didn't really have latent compartments, maybe cite Jessica Conway / Alison Hill and our group if you like

Thank you for that suggestion. We see that this could have been read as suggesting that the early Perelson models included latency, when our intention was rather to highlight modeling studies that inspired our own. We have now reworded this statement to be clearer and expanded our references.

-"reactivation dominates, suppressing" this makes it seem like the reactivation does something to proliferaiton? but you just mean that the reactivation is more common so net is negative right?

Yes, that is correct. We have now reworded this statement to be more clear.

-might need to explain that the levels in fig3 are the sum of all the clones

This is a good point. We have now made clear that these values are aggregated across all clones.

-I would love to see the clonal dynamics during fig3, it would be a super nice visual to color clones by reactivation rate and show the diversity decreasing but also the trend toward large but stable defective clones over time after ART

We have now added new **Supplementary Figs. 3, 5** and **7** that show different components of LR dynamics, including the distribution of clone ages before and after ART, the distribution of probabilities of reactivation before and after ART (showing the expected shift toward more quiescent clones), and dynamics of clone size.

-one thing Matthias Lictherfeld's group has shown also is that intact sequences can persist for a really long time if they are transcriptionally silenced (ie in specific integration sites). I think your model could be framed to explain this: occasional intact sequences that have very low reactivation probability -- in the model now this is assumed to be about Ag stimulation, but could encompass the other mechanism if you want to write about that in discussion

This is an excellent connection, thank you for the suggestion. Indeed, it seems plausible that clones with lower transcriptional activity may also be less likely to reactivate. Their finding of a progressive decrease in transcriptionally active clones over time also mirrors the decline in highly reactive clones in our model. We now discuss these references more explicitly in the Discussion.

-fig4B on reservoir seeding, maybe you could frame in terms of what you see is bursts not leading to "sequential" evolution, i.e. star-like vs ladder-like

Thank you for this suggestion. Indeed, we believe that this phenomenon of regular “bursts” of replication, as opposed to continual, self-sustaining (if low-level) replication, is important. We have added this phylogenetic intuition in the Results where **Fig. 4B** is described.

-relatedly, does your model make any predictions about the frequency of blips and unsuppressable viremia?

This is a very interesting question. In general, we do observe spikes of viral replication akin to blips in our simulations. Especially for phenomena such as unsuppressable viremia, however, we believe that more sophisticated models that explicitly incorporate features like host immune responses may be required. Very roughly, though, we would typically expect that the frequency of blips should be affected by the total size of the inducible reservoir (something like IUPM) and exposure to antigens that could stimulate bursts of reactivation.

-"mimic elite control or early ART" - two points on this: 1) I think EC is a lot more complicated than is handled here so it might be better to just focus on early ART, 2) to go a little further, it would be interesting to see if your simulated reservoirs with early ART also woudl rebound slower? I think there's something interesting going on here with the balance between reactivation probability but also few clones/small reservoir

Regarding elite control, this is a very reasonable point. We have removed our references to elite control and highlighted explicit modeling of host immune responses as an important model extension in the Discussion.

Although our model has not been calibrated to match the statistics of viral rebound in ATI trials, we performed simple, exploratory simulations to mimic the ATI process. In standard simulations, we “interrupted” ART five years after ART initiation by setting the viral infectivity parameter back to its pre-ART value. We then used the time between ATI and when the viral load crossed 200 copies per mL as the time to rebound. On average, we observed a time to rebound of around 10 days in standard simulations (see the new **Supplementary Fig. 11**), although we note that this depends on the choice of the infectivity parameter. In the early ART ATI simulations conducted 5 years after ART initiation, we observed a broad range in rebound times due to the small size of the reservoir. Thus, to obtain a tighter distribution, we simulated ATI one year after ART in early ART cases. Even in this scenario, we found that the time to rebound was longer (~26 days, **Fig. R10**). We have now added a discussion of these findings in our section “Effects of early intervention on LR structure,” with a description of the ATI simulations in Methods.

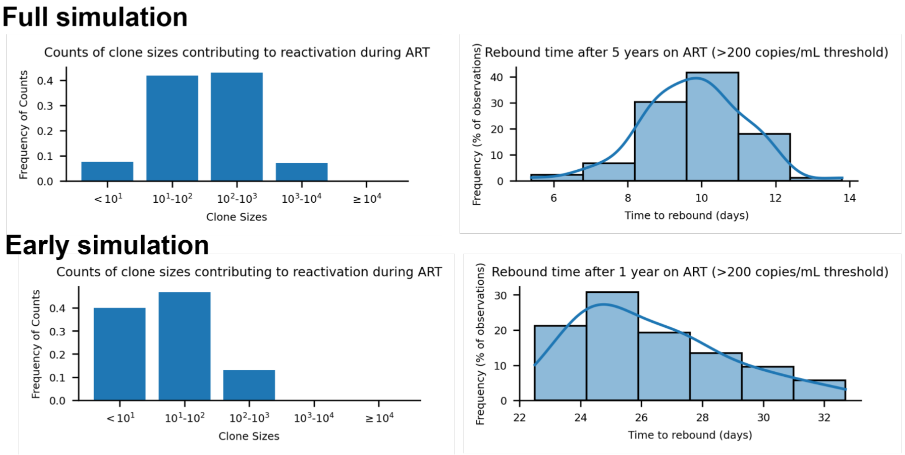

Fig. R10

-we've done some recent work to try to model both the HIV clones and the TCR clones that harbor them (https://www.medrxiv.org/content/10.1101/2024.02.13.24302704v1) it would be very nice to see your formulation extended eventually to see if it already captures the carrier cell dynamics too

We agree that this is a very interesting direction for future exploration, and we have added a reference to this work in the Discussion.

-there's also a nice paper on TCR clone modeling that might be contextualized/contrasted with your model and results: https://www.pnas.org/doi/10.1073/pnas.2207516120

Thank you for this suggestion as well. One aspect of this work that would be interesting to compare with our results (along with some studies in the specific context of the latent reservoir) is quantifying fluctuations in clone size over time. We have also referenced this work in the Discussion.

In closing, we appreciate the opportunity to revise our manuscript, and we are grateful to the reviewers for their constructive comments, which have helped to strengthen the paper.

Sincerely,

John Barton
Associate Professor
Department of Computational and Systems Biology
University of Pittsburgh School of Medicine
