# Supplementary material for: Clonal heterogeneity and antigenic stimulation shape persistence of the latent reservoir of HIV
Source: PLoS Comput Biol. 2025 Sep 15;21(9):e1013433. doi: 10.1371/journal.pcbi.1013433 (PMC12445745; doi:10.1371/journal.pcbi.1013433)
Supplement: Attachment — Submitted filename: PCOMPBIOL-D-24-01812_reviewer-comments.docx [file pcbi.1013433.s015.docx]

Noceda and Barton develop a stochastic model of HIV latent reservoir dynamics to investigate potential mechanisms by which clonal heterogeneity develops. Better understanding the development of clonal heterogeneity may provide insight into what types of treatments may be more likely to lead to an HIV cure. The authors show that their simulation fits well to real data and use their model to investigate the dynamics of clones over time during HIV infection pre-and post-ART initiation. While the results appear promising, the authors only show one simulation result, despite the model being stochastic, and do not always clearly show that the model supports the conclusions they make.

Major comments:

1. It appears that throughout the manuscript (Figures 2-5) only one simulation result is shown. However, the model is stochastic, which means that different simulation runs may lead to vastly different outputs. Therefore, the authors should run many different simulations (ideally ≥100 or 1000) and present the results of all the simulations, including a discussion of the variability across simulations, so the reader can better understand how consistent the results are.
2. One of the main conclusions, mentioned in the title, abstract, and discussion, is that antigenic stimulation and clonal heterogeneity shape the dynamics of the latent reservoir. In the model, latent cell activation and proliferation are dependent on both the probability of reactivation and the antigen levels. Therefore, it is difficult to differentiate between the effects of antigenic stimulation vs. different reactivation probabilities. To make claims about which of these is influencing LR dynamics, the authors must investigate the impact of each of these components not only together, but also independently. What do the results look like: (1) If simulations are performed where antigenic stimulation is constant across all cells and over time, but cells have different reactivation probabilities? (2) If simulations are performed with a single reactivation probability but fluctuating antigenic stimulation? (3) If both of these are held constant? Do these simulations have a better/worse/similar fit to the data?
3. Throughout the results, statements are made that appear reasonable but are not supported with numbers or figures. For example: (1) the viral load drop is primarily driven by the death of actively infected cells, (2) larger clones are slowly eliminated, (3) small clones with high probabilities of reactivation turn over frequently while large clones slowly fluctuate in frequency, (4) after a year on ART the relative size of large clones changes little over time. It is important to back up these types of statements with numbers/figures from the simulations.
4. You mention that “even though we find that the largest clones (with small probabilities of reactivation) are chiefly responsible for LR persistence, they may not be the typical first source of outgrowing viruses during rebound.” Can you simulate this to see if your hypothesis is true?
5. Related to clone size, reactivation rate, and age over time. It would be interesting to see the plots in Fig 2 and Fig 4 for times between 4 months and 60 months, and after 12 months on ART. It would also be helpful if all these plots were in one figure so that it is easier to see the progression over time. You also say that 1 year post-infection, the clones on average are young and likely to reactivate – how does this change throughout infection? It would be nice to include as a figure the distribution of clone ages at different times of infection.
6. In Fig 3, can you include the kinetics of plasma viral load, HIV-1 DNA, and the inducible viral reservoir pre-ART as well as post-ART?
7. The discussion of how the model output changes with respect to the lognormal distribution parameters should go in the main text rather than the methods. A supplemental figure supporting the statement would also be helpful.

Minor comments:

1. Why does Figure 3C start at 1 year post-ART when the others start at 0 years post-ART? I think it would make more sense to start all of them at 0. (Or, as mentioned above, also look at pre-ART levels.)
2. If you want to describe why you’re seeing the trends in Fig 3, then Fig 4A should be referenced there as well.
3. Could you put the bars for Fig 4B side-by-side so it is easier to see the difference in the accumulated number of mutation for 1 vs. 5 years? (e.g. make the years two different colors)
4. In the equations, what is W?
